# Health-related quality of life after laparoscopic and open surgery for rectal cancer in a randomized trial

**DOI:** 10.1002/bjs.9144

**Published:** 2013-05-03

**Authors:** J Andersson, E Angenete, M Gellerstedt, U Angerås, P Jess, J Rosenberg, A Fürst, J Bonjer, E Haglind

**Affiliations:** 1 Scandinavian Surgical Outcomes Research Group (SSORG), Department of Surgery, Sahlgrenska University Hospital/ÖstraGothenburg, Sweden; 2 Department of Surgery, Alingsås HospitalAlingsås, Sweden; 3 Department of Surgery, Roskilde HospitalRoskilde, Denmark; 4 Department of Surgery, Herlev Hospital, University of CopenhagenCopenhagen, Denmark; 5 Department of Surgery, Caritas Clinic St JosefRegensburg, Germany; 6 VUmc University Medical CentreAmsterdam, The Netherlands

## Abstract

**Background:**

Previous studies comparing laparoscopic and open surgical techniques have reported improved health-related quality of life (HRQL). This analysis compared HRQL 12 months after laparoscopic *versus* open surgery for rectal cancer in a subset of a randomized trial.

**Methods:**

The setting was a multicentre randomized trial (COLOR II) comparing laparoscopic and open surgery for rectal cancer. Involvement in the HRQL study of COLOR II was optional. Patients completed the European Organization for Research and Treatment of Cancer (EORTC) QLQ-C30 and QLQ-CR38, and EuroQol – 5D (EQ-5D™) before surgery, and 4 weeks, 6, 12 and 24 months after operation. Analysis was done according to the manual for each instrument.

**Results:**

Of 617 patients in hospitals participating in the HRQL study of COLOR II, 385 were included. The HRQL deteriorated to moderate/severe degrees after surgery, gradually returning to preoperative values over time. Changes in EORTC QLQ-C30 and QLQ-CR38, and EQ-5D™ were not significantly different between the groups regarding global health score or any of the dimensions or symptoms at 4 weeks, 6 or 12 months after surgery.

**Conclusion:**

In contrast to previous studies in patients with colonic cancer, HRQL after rectal cancer surgery was not affected by surgical approach. Registration number: NCT0029779 (http://www.clinicaltrials.gov).

## Introduction

There have been extensive studies of laparoscopic resection for colonic cancer, including randomized clinical trials showing short-term advantages for a minimally invasive approach. Some have also studied health-related quality of life (HRQL), reporting the superiority of laparoscopic surgery^1^,^2^. Laparoscopic surgery for rectal cancer has been studied less extensively. The impact of a permanent stoma on HRQL has been described, as has the change of HRQL over time in patients treated for rectal cancer^3^–^5^. In a prospective comparison of the effects of laparoscopic *versus* open surgery, Li and colleagues^6^ found improved HRQL 1 week after laparoscopic surgery, but not after 1 year. The present study compared HRQL 1 year after laparoscopic or open surgery for rectal cancer in a subset of patients from the international multicentre randomized clinical trial COlorectal cancer Laparoscopic or Open Resection (COLOR) II^7^.

## Methods

### The COLOR II trial

The patients in this HRQL study constituted a subset of the COLOR II trial cohort^7^. Thirty hospitals in eight countries (Belgium, Canada, Denmark, Germany, the Netherlands, South Korea, Spain and Sweden) participated in COLOR II, but inclusion in the HRQL study was optional. The primary endpoint of the trial is local recurrence rate, and the trial was designed as a non-inferiority study. Patients were randomized between laparoscopic and open surgery in the ratio 2 : 1, and the trial was stratified according to centre, preoperative radiation and type of operation. The inclusion criteria focused on selection of patients undergoing elective surgery for potentially curable rectal cancer, T1–T3, best treated by partial mesorectal excision, total mesorectal excision or abdominoperineal resection. Exclusion criteria included transanal resection. The protocol of the COLOR II trial was approved by the appropriate ethics committees^7^, and registered at http://ClinicalTrials.gov (NCT0029779).

### Patients

Twelve hospitals in five countries (Canada, Denmark, Germany, the Netherlands and Sweden) participated in the HRQL component of the COLOR II trial. Inability to understand the questionnaires was an exclusion criterion. Patients who agreed to participate were asked to complete the preoperative questionnaire within 5 days before the operation, then 4 weeks, 6, 12 and 24 months after surgery. In Dutch hospitals, patients were also asked to complete EuroQol – 5D (EQ-5D™; EuroQol Group, Rotterdam, The Netherlands) questionnaires 3, 7 and 14 days after operation. The results at 24 months will be published separately.

Demographic details, data on complications, tumour stage as classified in the pathology report on the resected specimen, reoperations, postoperative adjuvant chemotherapy, sexual function, and urinary and faecal continence, as recorded in clinical record forms at follow-up outpatient visits, were retrieved from the COLOR II database in Halifax, Canada. An analysis of sexual and urinary function will be presented separately, including European Organization for Research and Treatment of Cancer (EORTC) QLQ-PR25 together with data from clinical follow-up.

### Health-related quality-of-life instruments

The instruments used and reported here were EQ-5D™, EORTC QLQ-C30 and EORTC QLQ-CR38. Validated Swedish, Dutch, Danish, English and German translations of the instrument were used^8^.

#### EuroQol – 5D

The EQ-5D™ is a generic measure of health status. It is a standardized non-disease-specific (generic) instrument for assessing self-reported health status, allowing for comparisons across disease groups^9^. It comprises a description of the patient's health in five dimensions (mobility, self-care, daily activity, pain/discomfort and anxiety/depression). One of three levels is chosen for each dimension; the first level denotes no problems or a low level of symptoms, whereas the third level denotes severe problems or a high level of symptoms. Also included in the instrument is a vertical ‘thermometer’ (EQ-VAS) in which the patient is asked to assess their global health on a visual analogue scale from 0 (worst imaginable health state) to 100 (best imaginable). Respondents were requested to assess their health status on the day they filled out the questionnaire.

#### European Organization for Research and Treatment of Cancer QLQ-C30 and QLQ-CR38

The EORTC QLQ-C30 is a questionnaire developed to assess the quality of life of patients with cancer. The instrument available at the start of the study (2004) was version 3.0, a 30-item instrument designed for self-administration. The validated Swedish, English, Dutch, Danish and German translations were used^10^,^11^. This instrument has cross-cultural validity and the psychometric properties are considered satisfactory^12^. Normative data are available for German^13^ and Swedish^14^ patients as well as reference values^15^.

The QLQ-C30 questionnaire consists of 30 questions^16^. Both multi-item and single-item scales are constructed from the questions. There are five functional scales (physical, role, emotional, cognitive and social functioning), three symptom scales (fatigue, nausea/vomiting and pain), six single-item questions (about dyspnoea, insomnia, loss of appetite, constipation, diarrhoea and financial difficulties) and a global health/quality-of-life index. The latter assesses overall health and overall quality of life on a seven-point scale, where 1 indicating very poor and 7 indicating excellent. All other questions have four possible answers: ‘not at all’, ‘a little’, ‘quite a bit’ and ‘very much’. The time frame was ‘during the past week’.

The EORTC QLQ-CR38 questionnaire is used to measure more specific information about quality of life in patients with colorectal cancer. It is constructed in a similar manner to QLQ-C30. Thirty-eight questions cover four functional scales/single items (body image, sexual functioning, sexual enjoyment, future perspective) and eight symptom scales/items (micturition problems, chemotherapy side-effects, gastrointestinal symptoms, male sexual problems, female sexual problems, defaecation problems, stoma-related problems and weight loss). At the start of the study in 2004, QLQ-CR38 was available in the appropriate languages.

For both instruments individual scores were converted to a score ranging from 0 to 100, according to the EORTC scoring manuals. A high score for the symptom/item scales represents a high level of symptoms/problems, whereas a high score for the functional scales and the global health/general quality-of-life index represents a high level of functioning, overall health and quality of life.

### Statistical analysis

Because the study was piggy-backed on to a randomized trial with power calculated for the primary endpoint, no power calculation was performed for the HRQL component. Missing data were handled as instructed in the EORTC scoring manual. All statistical analysis of demographic data, relevant clinical outcome measures and differences between study groups was carried out using SPSS® 20 software (IBM, Armonk, New York, USA).

Comparisons of groups at baseline were made using Student's *t* test, χ^2^ test and, where appropriate, Fisher's exact test. EQ-5D™ global health was analysed at each assessment by means of the independent *t* test and repeated-measurement ANOVA was used for analysis over time. Proportions of patients reporting each level of the five dimensions were analysed by χ^2^ test or Fisher's exact test. As few patients reported problems at level 3 (severe problems), levels 2 and 3 were pooled in most analyses. QLQ-C30 and QLQ-CR38 global quality-of-life, functional and symptom scales were analysed using ANCOVA with baseline (preoperative score) as a co-variable and surgical procedure as a factor. The results are presented as mean changes, adjusted for baseline, with 95 per cent confidence intervals.

All statistical analyses were carried out on the basis of intention to treat. *P* < 0·050 was considered statistically significant. Owing to the explorative nature of this study, significant *P* values should be interpreted with care, and considered as interesting findings rather than conclusive evidence.

## Results

**Fig. 1:**
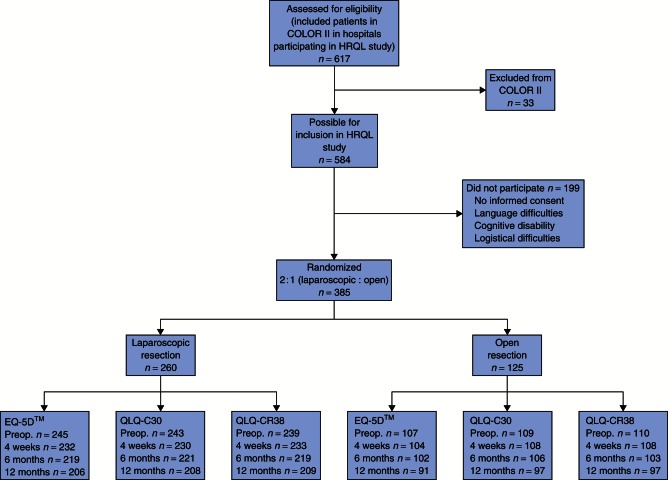
Study flow chart. HRQL, health-related quality of life; EQ-5D™, EuroQol – 5D

The COLOR II trial included 1103 patients between 2004 and 2010. In all, 617 patients were eligible for the HRQL study (*Fig*. 1). Thirty-three patients were excluded from the COLOR II trial after randomization as they did not conform to the inclusion criteria, and another 199 were primarily eligible but were not included owing to logistical difficulties in retrieving preoperative HRQL data, organizing preoperative radiation, language difficulties, patients' cognitive disabilities or lack of consent. Thus, 385 patients were included in the study (260 laparoscopic and 125 open). The included patients had a lower American Society of Anesthesiologists grade and fewer had undergone preoperative radiation compared with eligible patients who were not included. Basic demographic characteristics and clinical data did not differ between the laparoscopic and open groups (*Table* 1).

**Table 1:** Demographics for health-related quality-of-life study and for those not included in this study

	Not included in HRQL study (*n* = 199)	Included in HRQL study (*n* = 385)	*P*§	Laparoscopic (*n* = 260)	Open (*n* = 125)	*P*§
Age (years)*	67 (66·0, 69·4)	67·1 (66·1, 68·1)	0·696¶	67·4 (66·1, 68·6)	66·6 (64·8, 68·4)	0·487¶
Sex ratio (M : F)	123 : 76	239 : 146	0·949	162 : 98	77 : 48	0·893
Body mass index (kg/m^2^)*	25·9 (25·3, 26·5)	26·0 (25·6, 26·5)	0·750¶	26·0 (25·4, 26·6)	26·1 (25·3, 26·8)	0·898¶
ASA fitness grade						0·624
I	37 (18·9)	103 (26·8)	0·008	69 (26·5)	34 (27·2)	
II	101 (50·8)	224 (58·2)		149 (57·3)	75 (60·0)	
III	48 (24·1)	55 (14·3)		40 (15·4)	15 (12·0)	
IV	1 (0·5)	2 (0·5)		2 (0·8)	0 (0)	
Unknown	12 (6·0)	1 (0·3)		0 (0)	1 (0·8)	
Tumour stage†						0·552
I	8 (4·0)	22 (5·7)	0·262	18 (6·9)	4 (3·2)	
II	71 (35·7)	135 (35·1)		93 (35·8)	42 (33·6)	
III	101 (50·8)	207 (53·8)		135 (51·9)	72 (57·6)	
IV	7 (3·5)	12 (3·1)		9 (3·5)	3 (2·4)	
Unknown	12 (6·0)	9 (2·3)		5 (1·9)	4 (3·2)	
Type of resection						0·956
Partial mesorectal excision	15 (7·5)	42 (10·9)	0·632	27 (10·4)	15 (12·0)	
Total mesorectal excision	112 (56·3)	219 (56·9)		147 (56·5)	72 (57·6)	
Abdominoperineal excision	64 (32·2)	116 (30·1)		80 (30·8)	36 (28·8)	
Other	3 (1·5)	6 (1·6)		4 (1·5)	2 (1·6)	
Unknown	5 (2·5)	2 (0·5)		2 (0·8)	0 (0)	
Preop. radiotherapy‡	133 (66·8)	216 (56·1)	0·001	150 (57·7)	66 (52·8)	0·409
Short	110 (55·3)	157 (40·8)		110 (42·3)	47 (37·6)	
Long	23 (11·6)	40 (10·4)		27 (10·4)	13 (10·4)	
None	60 (30·2)	168 (43·6)		110 (42·3)	58 (46·4)	
Unknown	6 (3·0)	1 (0·3)		0 (0)	1 (0·8)	
Preop. chemotherapy			0·895			0·816
Yes	31 (15·6)	64 (16·6)		44 (16·9)	20 (16·0)	
No	145 (72·9)	290 (75·3)		195 (75·0)	95 (76·0)	
Unknown	23 (11·6)	31 (8·1)		21 (8·1)	10 (8·0)	
Conversion	–	–		65 (25·0)	–	

Values in parentheses are percentages unless indicated otherwise;

* values are mean (95 per cent confidence interval).

† Stage in the pathology report of the resected specimen.

‡ Short regimen comprised 5 × 5 Gy or less, and long programmes more than 5 days. Among those included in the health-related quality-of-life (HRQL) study, the dose specification was missing for 13 patients (5·0 per cent) in the laparoscopic group and six (4·8 per cent) in the open group. ASA, American Society of Anesthesiologists.

§ χ^2^ test, except

¶ Student's *t* test.

The intention was to analyse the change in HRQL from baseline (preoperative data) over time and compare the groups. Analysis of stoma-related problems was therefore excluded from this part of the study. The actual results at 4 weeks, 6 months and 12 months regarding these problems, with comparisons between groups, are presented, but for obvious reasons without comparison with preoperative data (see *Table* 5).

Compliance in answering the questionnaires was generally around 90 per cent at baseline and diminished over time to around 80 per cent at 12 months (*Fig*. 1). The compliance for EQ-5D™ was lower than this in the open group, being around 80 per cent at baseline and 70 per cent at 12 months. Compliance with the EQ-5D™ global health part was lower than for EQ-5D™ dimensions or EORTC questionnaires. For EORTC QLQ-C30 and QLQ-CR38 the answer rates were between 88 and 85 per cent at 4 weeks and 6 months, and 76–78 per cent at 12 months.

There were no significant differences between the two groups at any time in overall health measured by EQ-5D™ (*Table* 2), nor was the repeated-measurement analysis significant (*P* = 0·171–0·966). Regarding the five dimensions, the only significant difference was in ‘daily activity’; a higher proportion of patients in the open group reported problems before treatment (level 2–3) (*Table* 3).

**Table 2:** EuroQol – 5D global health scores

	Preop.	4 weeks	6 months	12 months
Mean(s.d.) EQ-5D™ score				
Laparoscopic	77·3(16·6)	64·2(20·8)	77·5(16·2)	79·4(15·9)
Open	74·9(16·6)	62·6(20·4)	75·7(18·0)	78·7(15·1)
Mean change*	2·4 (−1·5, 6·3)	1·6 (−3·3, 6·5)	1·7 (−2·4, 5·9)	0·6 (−3·4, 4·7)
*P*†	0·228	0·981	0·815	0·646

* Values in parentheses are 95 per cent confidence intervals. EQ-5D™, EuroQol – 5D.

† Independent *t* test.

**Table 3:** Results for the five health dimensions of the EuroQol – 5D

	% of patients							
	
	Preop.		4 weeks		6 months		12 months	
				
	Laparoscopic	Open	Laparoscopic	Open	Laparoscopic	Open	Laparoscopic	Open
Mobility								
Level 1	91	88	68	65	85	82	87	88
Level 2	9	12	30	34	14	18	13	11
Level 3	0	0	2	1	1	0	0	1
Self-care								
Level 1	99	98	87	85	96	92	96	97
Level 2	1	2	12	13	4	7	4	3
Level 3	0	0	1	2	0	1	0	0
Daily activity								
Level 1	89	80	40	37	73	72	80	80
Level 2	10	15	43	48	24	23	18	17
Level 3	1	5	17	15	3	5	2	2
Pain/discomfort								
Level 1	58	49	28	30	49	53	59	55
Level 2	40	49	66	68	49	44	40	44
Level 3	2	2	6	2	2	3	1	1
Anxiety/depression								
Level 1	60	51	55	52	68	68	72	68
Level 2	37	42	42	47	29	32	26	32
Level 3	3	7	3	1	3	0	2	0

Level 1, no problems; level 2, low level of symptoms; level 3, severe problems or high level of symptoms. The only significant difference between groups was in daily activity before treatment (*P* = 0·024, χ^2^ test).

HRQL measured by the cancer-specific EORTC QLQ-CR30 showed no statistically significant differences between groups in any dimension (global quality of life, five functional scales and three symptom scales) either before, or 4 weeks, 6 months and 12 months after surgery (*Table* 4). There were changes in most functional scales and symptoms between baseline and 4 weeks after surgery within both treatment groups. Global quality of life was restored by 12 months after both types of surgery, as were scores on most functional scales and symptoms, whereas emotional function had improved by 12 months.

**Table 4:** Changes in function and symptom scores on European Organization for Research and Treatment of Cancer QLQ-C30

		4 weeks		6 months		12 months	
				
	Mean preop. score	Mean change from preop.	Adjusted mean difference (laparoscopic – open)	Mean change from preop.	Adjusted mean difference (laparoscopic – open)	Mean change from preop.	Adjusted mean difference (laparoscopic – open)
Global quality of life*							
Laparoscopic	72·8 (70·2, 75·3)	−14.8	0·3 (−4·7, 5·3)	−1.9	−2·2 (−6·8, 2·4)	2.1	−1·8 (−6·1, 2·4)
Open	68·6 (64·7, 72·6)	−11.9		3.0		6.3	
Physical function*							
Laparoscopic	88·7 (86·8, 90·6)	−21.6	0·2 (−4·8, 5·1)	−6.7	−0·5 (−3·8, 2·8)	−3.4	0 (−3·1, 3·0)
Open	88·8 (86·0, 91·5)	−21.6		−6.0		−3.3	
Role function*							
Laparoscopic	80·9 (77·5, 84·4)	−34.9	−1·7 (−9·0, 5·6)	−4.9	1·7 (−4·4, 7·6)	−0.8	−0·9 (−6·4, 4·6)
Open	81·9 (76·8, 87·0)	−33.7		−7.7		0.6	
Emotional function*							
Laparoscopic	77·2 (74·4, 80·0)	−2.5	−1·7 (−6·5, 3·0)	6.1	−2·4 (−6·4, 1·6)	7.1	−2·7 (−7·1, 1·6)
Open	74·2 (70·1, 78·3)	1.2		10.2		12.0	
Cognitive function*							
Laparoscopic	88·9 (86·8, 91·0)	−8.4	−1·2 (−6·2, 3·7)	−0.8	3·5 (−0·1, 7·1)	−1.2	0 (−3·9, 3·8)
Open	89·3 (86·5, 92·0)	−7.1		−4.2		−0.8	
Social function*							
Laparoscopic	87·0 (84·4, 89·5)	−22.4	−0·4 (−7·0, 6·2)	−8.1	0 (−5·5, 5·5)	−3.1	0·7 (−4·7, 6·1)
Open	84·4 (80·4, 88·5)	−20.7		−7.1		−2.4	
Fatigue†							
Laparoscopic	22·8 (19·8, 25·7)	25.0	2·1 (−3·8, 8·0)	5.2	−1·0 (−6·1, 4·0)	0.5	−0·1 (−4·5, 4·3)
Open	25·8 (21·9, 29·6)	21.0		4.7		−1.6	
Nausea and vomiting†							
Laparoscopic	4·9 (3·3, 6·5)	2.7	−2·8 (−6·5, 1·0)	−1.4	−1·0 (−3·8, 1·9)	−2.6	1·2 (−0·6, 2·9)
Open	4·1 (2·4, 5·9)	6.0		0.4		−3.3	
Pain†							
Laparoscopic	14·3 (11·6, 17·1)	18.5	−0·1 (−6·5, 6·3)	2.3	1·8 (−3·1, 6·8)	0.3	0·1 (−4·3, 4·4)
Open	13·9 (9·9, 17·9)	18.5		0.4		−1.1	
Dyspnoea†							
Laparoscopic	10·8 (8·4, 13·2)	10.0	−0·1 (−6·3, 6·1)	5.6	2·5 (−2·3, 7·3)	3.5	3·0 (−1·6, 7·7)
Open	12·8 (8·2, 17·3)	9.4		1.8		−0.8	
Insomnia†							
Laparoscopic	26·1 (22·5, 29·8)	4.5	−3·9 (−10·9, 3·0)	−5.0	0·7 (−4·8, 6·3)	−6.4	2·3 (−2·9, 7·5)
Open	26·8 (21·6, 32·0)	8.1		−5.8		−9.8	
Appetite loss†							
Laparoscopic	9·7 (7·0, 12·5)	17.1	−4·3 (−11·7, 3·2)	−2.3	−2·0 (−6·7, 2·8)	−3.9	1·8 (−1·8, 5·4)
Open	10·3 (6·3, 14·2)	21.1		−0.4		−6.6	
Constipation†							
Laparoscopic	12·8 (9·7, 16·0)	−2.4	−0·7 (−5·9, 4·5)	−4.5	−0·8 (−5·3, 3·6)	−5.1	0·3 (−4·1, 4·7)
Open	9·3 (5·2, 13·4)	1.0		1.1		−1.6	
Diarrhoea†							
Laparoscopic	27·1 (23·0, 31·2)	−11.0	2·5 (−3·5, 8·6)	−8.1	1·6 (−4·9, 8·1)	−7.9	6·0 (−0·4, 12·4)
Open	30·5 (24·2, 36·9)	−17.9		−15.1		−18.4	
Financial difficulties†							
Laparoscopic	6·7 (4·0, 9·4)	4.4	−1·2 (−6·0, 3·7)	2.0	−0·4 (−5·1, 4·3)	−1.3	−2·1(−5·7, 1·6)
Open	4·7 (1·5, 7·8)	6.2		3.1		2.1	

Values in parentheses are 95 per cent confidence intervals.

* A high value is positive to the patient;

† a high value is negative to the patient.

**Table 5:** Changes in scores on European Organization for Research and Treatment of Cancer QLQ-CR38

		4 weeks		6 months		12 months	
				
	Mean preop. score	Mean change from preop.	Adjusted mean difference (laparoscopic – open)	Mean change from preop.	Adjusted mean difference (laparoscopic – open)	Mean change from preop.	Adjusted mean difference (laparoscopic – open)
Body image†							
Laparoscopic	90·3 (88·2, 92·4)	−17.8	0·4 (−5·9, 6·6)	−13.8	−2·0 (−7·9, 3·9)	−11.5	−2·8 (−8·7, 3·0)
Open	87·4 (83·8, 90·9)	−17.1		−10.1		−6.6	
Future perspective†							
Laparoscopic	57·1 (53·5, 60·7)	5.6	2·0 (−4·5, 8·4)	10.2	−2·4 (−8·6, 3·8)	11.8	−2·7 (−8·9, 3·6)
Open	54·0 (48·2, 59·8)	5.7		14.3		16.7	
GI symptoms‡							
Laparoscopic	17·6 (15·7, 19·4)	6.9	2·6 (−1·1, 6·3)	−0.6	0·5 (−2·7, 3·7)	−0.8	0·1 (−3·0, 3·2)
Open	17·1 (14·6, 19·6)	4.5		−0.5		−1.1	
Defaecation problems‡							
Laparoscopic	26·5 (24·1, 29·0)	7.2	2·7 (−5·7, 11·0)	2.4	5·9 (0·2, 11·6)	−1.2	4·2 (−0·4, 8·7)
Open	26·0 (22·2, 29·8)	6.7		−3.4		−5.8	
Weight loss‡							
Laparoscopic	14·7 (11·7, 17·7)	22.8	−3·7 (−11·2, 3·9)	−0.7	−1·6 (−7·3, 4·2)	−5.6	1·6 (−2·8, 6·0)
Open	14·5 (10·2, 18·9)	26.5		−0.4		−8.0	
Chemotherapy side-effects‡							
Laparoscopic	8·8 (7·2, 10·5)	13.8	−0·9 (−5·8, 4·0)	6.8	−0·8 (−5·0, 3·5)	2.7	0 (−3·7, 3·6)
Open	10·5 (7·3, 13·7)	13.5		6.6		1.3	
Stoma-related problems*‡							
Laparoscopic	–	30.6	−1·0 (−6·7, 4·7)	25.2	−4·8 (−11·0, 1·4)	27.5	−1·3 (9·4, 6·7)
Open	–	31.6		30.0		28.8	

Values in parentheses are 95 per cent confidence intervals.

* Only six patients had a stoma before surgery. Values at 4 weeks, 6 months and 12 months are mean scores instead of mean change in score.

† A high value is positive to the patient;

‡ a high value is negative to the patient. GI, gastrointestinal.

There were no differences between groups in EORTC QLQ-CR38 data at any time point measured (*Table* 5). Future perspective scores improved over time in both groups, with no difference between the two surgical techniques.

## Discussion

This study has shown no difference in the changes to HRQL within 12 months after laparoscopic and open surgery for rectal cancer. It is important to evaluate what constitutes a clinically significant difference. In regard to EORTC QLQ-C30, several studies have examined the minimal important change (MID) implicating a change that is clinically meaningful to the patient. Osoba^17^ has suggested that the MID is in the range of 5–10 points on the 100-point scale, whereas over 20 points indicates a substantial change. In the present study, the changes reported for most functional scales and symptoms, in both the EORTC QLQ-C30 and QLQ-C38, were substantial or moderate after 4 weeks, and gradually diminished over time. All results were within narrow confidence intervals, which supports the validity of the results, and also excludes any ‘clinically relevant’ differences between the groups.

Physical functioning, role functioning, social function and fatigue measured by QLQ-C30 showed substantial deterioration 4 weeks after surgery. All of these functional/symptom scales improved after 6 months and were fully recovered at 12 months. The time frame differed from that in laparoscopic surgery for colonic cancer, where physical function and role function were reduced after 2 weeks, but partially recovered within 4 weeks^1^,^2^. It appears that patients with rectal cancer require a longer time to recover after curative surgery.

There was a selection bias in the present study cohort as participants were somewhat healthier in general than the entire COLOR II trial cohort. This could be the result of logistics related to radiotherapy treatment. For patients with a high level of co-morbidity the ability and/or inclination to answer questionnaires might be reduced. This was, however, true for both groups and the authors suggest that the lack of difference between laparoscopic and open surgery is valid.

There is no obvious explanation for the difference in compliance between the laparoscopic and open groups at baseline (*Fig*. 1). It is also intriguing that the compliance varied for the different instruments as they were sent out as a complete booklet at each time point. In particular, compliance in completion of EQ-5D™ at baseline differed, with lower compliance in the open group. The trial was not blinded so the patients were aware of which technique they had been randomized to. It could be speculated that, having agreed to participate in a randomized trial testing a new and presumably less invasive surgical technique, patients would be more ‘positive’ to the new technique and so those randomized to laparoscopy would also comply with the demands of this substudy. Baseline clinical data in the two groups were similar and, if the difference in compliance had represented a systematic difference in recruitment, differences in the results would have been expected. It is therefore argued that this difference most probably arose by chance.

HRQL assessment is important when evaluating new treatments. Patients today have a longer life expectancy, and the overall improved results of rectal cancer treatment, with 5-year survival rates of more than 60 per cent, indicate that there will be many survivors. The present results are therefore of interest as they reflect patients' experience after rectal cancer surgery. As the surgical technique resulted in no difference in HRQL, other factors, such as reduction in the risk of small bowel obstruction^18^,^19^ or the amount of perioperative bleeding or postoperative pain^20^, may influence the choice of surgical technique for rectal cancer.

The fact that HRQL after rectal cancer surgery is substantially reduced for a prolonged period is noteworthy, indicating the need for a high level of healthcare support for several months after operation. This is in agreement with the finding of Wilson and co-workers^21^, who reported that HRQL was impaired for up to 6 months after rectal cancer surgery. The present study showed clinically meaningful changes at 4 weeks after surgery, regardless of the surgical technique and for most functional scales, but these returned to, or were close to, preoperative values by 6 months. The findings in this HRQL study do not mirror the improved short-term clinical outcomes reported after laparoscopic colonic surgery, such as reduced pain and earlier restoration of bowel function. This could possibly be explained by the time points chosen for HRQL measurements, the first of the questionnaires being completed at 4 weeks after operation.

A previous study of patients who had surgery for inflammatory bowel disease found that body image was rated more highly after laparoscopic than open surgery^22^. This was not demonstrated here and, although speculative, body image may have been less important to the older patients in this trial.
